# Socio-demographic correlate of knowledge and practice toward COVID-19 among people living in Mosul-Iraq: A cross-sectional study

**DOI:** 10.1371/journal.pone.0249310

**Published:** 2021-03-31

**Authors:** Balsam Qubais Saeed, Rula Al-Shahrabi, Obasanjo Afolabi Bolarinwa

**Affiliations:** 1 Department of Clinical Sciences, College of Medicine, University of Sharjah, Sharjah, United Arab Emirates; 2 Sharjah Institute for Medical Research, University of Sharjah, Sharjah, United Arab Emirates; 3 Department of Public Health Medicine, School of Nursing and Public Health, University of KwaZulu-Natal, Durban, South Africa; Georgia Southern University, UNITED STATES

## Abstract

Since the World Health Organization (WHO) announced that the coronavirus disease (COVID-19) is a worldwide pandemic, many countries’ authorities, including the Iraqi authorities, started responding and taking action to control the spread of the pandemic. The public’s knowledge and practices play an important role in curbing the spreading of the virus by following the health guidelines. This study aimed to assess the socio-demographic correlate of knowledge and practices of Iraqi living in Mosul-Iraq towards COVID-19 during its rapid rise. A cross-sectional online survey of 909 participants was conducted among a sample of the Mosul-Iraq population between 20^th^ June to 1^st^ July 2020. The survey included three parts: 1) socio-demographic characteristics, 2) participants’ knowledge, 3) participants’ practices. T-test, ANOVA, chi-square, and binary logistic regression were used. A p-value less than 0.05 (p < 0.05) was considered statistically significant. The results showed a knowledge and practice mean score of (12.91±1.67) and (21.56± 2.92) with cumulative knowledge and practice of 86% and 76% respectively towards COVID-19. Socio-demographic characteristics such as age, marital status, gender, level of education and employment were statistically related with a higher mean score of knowledge and practice towards the virus as *P<0*.*05*. We concluded that the majority of the respondents demonstrate a high level of knowledge and practices towards COVID-19 except for respondents with socio-demographic characteristics such as those who were younger, male respondents, those with lower education and those unemployed as such campaigns that will increase the knowledge and encourage adequate preventive practice towards COVID-19 should be targeted towards this group.

## Introduction

On 31^st^ December 2019, the 2019 coronavirus (COVID-19) detected in Wuhan, China, on 11-March 2020, has been considered a global pandemic by the World Health Organization (WHO), as at the end of April, the virus has spread worldwide with fear-evoking death reports [1, 2].

The severe acute respiratory syndrome coronavirus 2 (SARS-CoV-2) is highly contagious; it causes a respiratory illness that ranges from the common cold-like symptoms to more severe diseases [2]. Most of the infected patients complained of fever, shortness of breath, cough, loss of smell, and/or taste sensation and might be infected asymptomatically. In severe cases, patients might suffer from pneumonia, multiple organ dysfunction, and death [3, 4].

The SARS-CoV-2 is a novel strain that is not specified in humans earlier. It is a zoonotic illness that is transmitted between animals and people [5].

In Iraq, the first case of COVID-19 was confirmed on 24^th^ February 2020 in Al-Najaf city by people who have visited Iran. Therefore, at the end of March 13–2020, the Ministry of Health and Environment of Iraq declared that the total confirmed COVID-19 cases were 101 [6]. The ministry of health (MOH) in Iraq started to respond and control the infection with the technical observation of the WHO center, three governmental biological laboratories in Baghdad, Basrah, and Erbil, opened for COVID-19 outbreak testing [7]. Initially, the measures that were taken by the Iraqi authorities nationwide have succeeded in managing to slow the spread of the virus, but the cases have risen sharply in the beginning of July 2020. In the first week of August 2020, Mosul city recorded thirty times an increase in COVID-19 cases reported compared to the previous months, while the reported national cases rose from 10,000 to 120,000 across the country [8].

Due to the escalating number of cases in Mosul city, many challenges were observed while trying to stop the virus from spreading, the limited number of tests that can be carried out per day, lack of quarantine facilities, medical instrument, hygienic preparation insufficiency, low hospital capacities, and no approved medicine or vaccine to prevent the COVID-19. Therefore, people’s preventive measures are important to protect themselves and others from the virus infection and control the spread of the disease [9]. Thus, managing this crisis hangs primarily on people’s knowledge and practices toward this virus and follows all the precautions to prevent cross-infection and follow the WHO and the Center for Disease Control and Prevention (CDC) [10, 11].

WHO declared precautional strategies to curb the spreading of infections. The none medical precautions are maintaining social distancing, avoiding public gatherings, avoiding direct contact with infected people, and using personal protective equipment (PPE) like face masks. Personal hygiene recommendations such as hand-washing often with soap and water for at least 20 seconds, especially after touching surfaces, which also include an advice not to touch the nose, eyes, and mouth with unwashed hands, and self-isolate when COVID-19 symptoms started [10].

The knowledge and practices of the public toward COVID-19 play an important role in determining the willingness of Iraqi citizens to change their behavior and identify the kind of intervention that is needed to correct the misconceptions regarding the virus, highlight the poor knowledge toward the virus and disease, development of new preventive measures, develop COVID-19 awareness campaign, and take precautionary. Therefore, the current study aimed to assess the socio-demographic correlate of knowledge and practices of Iraqi living in Mosul towards Covid-19 during its rapid rise.

This study’s results are expected to provide baseline information about Iraqis’ level of knowledge and practices living in Mosul and highlight misperceptions related to preventive measures. The study outcomes will further plan for effective awareness campaigns, required interventions, and appropriate action from local authorities.

## Materials and methods

### Study design

A convenient cross-sectional survey was adopted for the study, the researchers use an online survey using "google forms" platform. Mosul-Iraqi individuals above the age of 18 and currently residing in Mosul were able to participate in the survey. The professional and personal networks of the researchers were used to reach a high number of participants to complete the survey. The standardized general invitation letter with the link were sanded to respondents to participate in this study by. by using the most popular communication and social platforms in Iraq (WhatsApp and, Facebook Messengers).

The questionnaire link was posted among specific Facebook groups, the members who clicked on the link were directed to the Google forms and to reduce the missing data, the participants were requested to fill all the questions of the questionnaire or else could not proceed to the next section. after complete, the answer to all questions the participant was directed to clicks on submit. The google based questionnaire was designed in English and Arabic languages in order to encourage the respondents’ adequate participation since Arabic is the common language in Mosul city.

### Study settings

Mosul is located in Nineveh Governorate—northern Iraq has a population of 664,221 as of 2015. Mosul city residents suffer from a fragile health system that barely meets their basic needs, as many health facilities were destroyed in 2017 [8].

### Survey and data collection

A total of 909 Mosul–Iraqi participated in our study between 20^th^ June to 1^st^ July 2020. The specified Sample size was determined by adopting the minimum acceptable size of a demographic subgroup with a ±5% margin of error and a confidence level of 95% [12, 13]. Giving to this, out of the total respondents of 1121 who filled the online google based questionnaire, only 909 were included. An incomplete survey of participants of 212 was excluded from the study, leaving us with a completion rate of 81%.

### Questionnaire design

The survey was an adapted version of questionnaires published previously [14–16]. The questionnaire was reviewed, validated and pilot tested by 21 Iraqi people and 3 faculty experts at the University of Mosul using WhatsApp and telephone interviews to correct any question before sending it to the target population and is it’s attached as a (S1 Questionnaire) in this study. The pilot test result was not included in the study results. The questionnaire consisted of an interface page and three main themes, with a total of 37 questions. The interface page included the title, objective of the study, information on participants’ privacy, and instructions to fill the survey. The three main themes included: 1) demographic information of participants such as gender, age, education level, marital status, employment status, coronavirus test, coronavirus result and Chronic diseases; 2) Knowledge related to the COVID-19 consisted of 15 questions divided into clinical presentations of virus (K1-K5), the spread of the virus (K6-K9) prevention (K10-K12) and the risk factors (K13-K15); 3) practices of participants toward COVID-19 outbreak, which included 13 questions.

### Ethical considerations

The Research Ethics Committee (RIC) at the University of Sharjah, UAE approved this study by the reference number is REC- 20-05-31-01, as of 14/06/2020. This approval also suffices for the surveys in Mosul, Iraq, since participation was voluntary and anonymous. Informed consent was sought from all the respondents prior to data collection. The participant who declined consent were not permitted to participate in the survey, and participants could withdraw from the survey at any time in line with stipulations of the World Medical Association Declaration of Helsinki Ethical principles [17].

### Data and statistical analysis

Data analysis conducted using Statistical Package for Social Software (SPSS) version 22. Scale reliability was performed to ensure data consistency in knowledge and practice aspects (included 15 questions of knowledge, and 14 practice questions) using Cronbach’s alpha coefficient with total reliability of 0.729, indicating good consistency [18]. Since the knowledge part measures four aspects which are clinical presentations, the spread of the virus, prevention, and the risk factors, internal consistency showed significance <0.001 in the correlation between these components and total knowledge. The frequencies of demographic characteristics were presented in frequency (number\percent), while knowledge, and practice answers, along with descriptive statistics, were presented in frequency, and mean ± SD. Participants’ knowledge and practice scores were compared with demographics factors using independent- samples t-test, one-way analysis of variance (ANOVA).

To measure the knowledge, participants were given “yes,” “no,” and “not sure” answer options to each survey question. A true answer to each question was marked with 1 score, while false answers and not sure were marked with 0 scores. Scores of total knowledge ranged from 0–15; a higher score signals a better knowledge level. To practice measures, participants were given "always", "sometimes", and " never” answer options to each item; the always option was marked for 2 scores, while sometimes was for 1 score, and rarely was for 0 scores. The total practice scores ranged from 0–26. The lowest and highest score of participants’ knowledges was 7 and 15, respectively, while the lowest and highest score of practice was 14 and 26, respectively.

Pearson’s chi-square was used to determine the association between the explanatory and outcome variables. We examined the factors associated with good knowledge & practice and poor knowledge & practices using binary logistic regression analyses. A p-value of less than 0.05 (p < 0.05) was considered statistically significant.

To identify the significantly associated factors with good or poor knowledge and practices, a mean knowledge score of more than 12 indicated as good knowledge, while less than 12 assigned as poor. Similarly, a mean practice score above 21 indicated good practice and a mean practice score below 21 as poor practice. The values have been determined by selecting a cut point of knowledge and practice based on the total mean score for all participants, The cut point 12 for knowledge was chosen as the total mean score for knowledge was 12.9, any scores below 12 considered poor according to each participant score. Similarly, with practice, the total mean score was 21.56, The cut point was 21. Factors were selected with a backward stepwise method, and the reference category was selected based on the higher total mean. Unstandardized regression coefficients (β) and odds ratios (ORs) and their 95% confidence intervals (CIs) were used to quantify the associations between cofactors with knowledge and practice.

## Results

### Socio-demographic characteristics

Table 1 showed that the majority of 558 (61.4%) were females, and 351 (38.6%) were males. More than half of the participants, 495 (54.5%), aged 30–49 years. Around 549 (60.4%) of respondents were married, while 288 (31.7%) and 72 (7.9%) were single, and others (divorced and widows), respectively. About 567 (62.4%) were holding a bachelor’s degree, while 234 (25.7%), 108 (11.9%) were holding postgraduates and diploma or below, respectively. Moreover, almost 585 (64.4%) were employed, while a smaller number of participants, 198 (21.8%), 126 (13.9%), were unemployed and students, respectively.

**Table 1:** Socio-demographic characteristics of the participants Mosul, Iraq (n = 909).

Demographic factors		n (%)
Gender	Female	558 (61.4%)
	Male	351 (38.6%)
Age	18–29	252 (27.7%)
	30–49	495 (54.5%)
	≥ 50	162 (17.8%)
Marital status	Single	288 (31.7%)
	Married	549 (60.4%)
	Others	72 (7.9%)
Education level	College degree	567 (62.4%)
	Postgraduate degree	234 (25.7%)
	Others	108(11.9%)
Employment status	Employed	585 (64.4%)
	Unemployed	198 (21.8%)
	Student	126 (13.9%)

### Chronic conditions reported by participants

In our study, 82.4% of the participants were healthy, while 17.6% had chronic diseases. The most common chronic diseases were diabetes 6%, followed by asthma at 3.30%. Moreover, 2.75%, 2.40%, 1.54%, 1.54%, 1.20% had chronic kidney disease, severe obesity, heart conditions, chronic liver disease, and chronic lung diseases, respectively, as shown in Table 2.

**Table 2:** Chronic conditions reported by participants in Mosul, Iraq (n = 909).

Chronic conditions	Frequency	%
Diabetes	52	6%
Asthma	30	3.30%
Severe obesity	22	2.40%
Chronic kidney disease	25	2.75%
Heart conditions	14	1.54%
Chronic liver disease	14	1.54%
Chronic lung diseases	11	1.20%

### Knowledge towards COVID-19

The mean knowledge score (±SD) of 15 questions was (12.91±1.67). The correct percentage rate was 86.08%. Most of the participants, 92.1%, answered correctly that COVID-19 was caused by a virus; the majority, 94.1%, knew that the incubation period range of this virus is between 2–14 days. Almost all participants had a high knowledge of 96% about the symptoms of a COVID-19; about 72.3% of respondents knew that no vaccine for this virus. Moreover, 63.4% knew that no treatment was approved for COVID-19 until now. When we asked if this virus is spread via respiratory droplets of infected people, 93.1% answered correctly.

Similarly, A high proportion of 93.1% of the participants agreed that the virus could be transmitted via touching contaminated surfaces. Also, 82.2% indicated that this virus was transmitted through the eyes, nose, and mouth. Just over half, 59.4%, reported that the infected person having no fever could infect healthy people and the majority of participants, 94.1% recorded that children and young adults have to take measures toward COVID-19.

About 76.2% of respondents reported the individuals should stay at home and go out only when necessary, while all of the participants 100% agreed that they should avoid going to crowded places, and the infected person with this virus should be immediately isolated in a proper place. Most of the respondents, 96%, answered that the virus is more dangerous for those with chronic disease patients and the elderly, while 79.2% believed that smokers are more vulnerable to this virus. The knowledge of participants toward COVID-19 is displayed in Table 3.

**Table 3:** General knowledge of participants about COVID-19 in Mosul, Iraq (n = 909).

Knowledge questions		Frequency (%)	MEAN±SD
K:1 COVID-19 is caused by the virus	**Yes**	**837 (92.1)**	0.92± 0.27
	No	27 (3.0)	
	Not sure	45 (5.0)	
K:2 Incubation period range of COVID-19 is 2–14 day	**Yes**	**855 (94.1)**	0.94±0.24
	No	45 (5.0)	
	Not sure	9 (1.0)	
K3: The main clinical symptoms of COVID-19 are fever, dry cough, tiredness, and breathing difficulties	**Yes**	**873 (96.0)**	0.96 ± 0.19
	No	0(0)	
	Not sure	36 (4.0)	
K4: No vaccine against COVID-19	**Yes**	**657 (72.3)**	0.72 ±0.45
	No	81 (8.9)	
	Not sure	171 (18.8)	
K5: No active treatment for COVID-19	**Yes**	**576 (63.4)**	0.64 ±0.48
	No	63 (6.9)	
	Not sure	270 (29.7)	
K6: COVID-19 spreads via respiratory droplets of infected people	**Yes**	**846 (93.1)**	0.93 ±0.25
	No	9 (1)	
	Not sure	54 (5.9)	
K7: COVID-19 is spread through touching contaminated surfaces	**Yes**	**846 (93.1)**	0.93 ±0.25
	No	9 (1)	
	Not sure	54 (5.9)	
K8: COVID-19 can be transmitted through eyes, in addition to nose and mouth	**Yes**	**747 (82.2)**	0.83 ±0.38
	No	108 (11.9)	
	Not sure	54 (5.9)	
K9: A person with COVID-19 having no fever cannot infect others	**Yes**	**162 (17.8)**	0.59±0.49
	No	540 (59.4)	
	Not sure	207 (22.8)	
K10: Children and young adults don’t need to take measures to prevent the infection by the COVID-19	**Yes**	**18 (2)**	0.94 ±0.24
	No	855 (94.1)	
	Not sure	36 (4)	
K11: We should stay at home and go out only when it is necessary	**Yes**	**693 (76.2)**	0.96 ±0.20
	No	216 (23.8)	
	Not sure	0 (0)	
K12: To prevent the spread of COVID-19, individuals should avoid going to crowded places if it’s not necessary	**Yes**	**909 (100)**	1.00 ±0.00
	No	0(0)	
	Not sure	0(0)	
K13: The COVID-19 may be more dangerous in patients with chronic diseases and elderly	**Yes**	**873 (96)**	0.79±0.41
	No	9 (1)	
	Not sure	27 (3)	
K14: People who have contact with someone infected with the COVID-19 should be immediately isolated in a proper place	**Yes**	**909 (100)**	1.00 ±0.00
	No	0(0)	
	Not sure	0(0)	
K15: Smokers are likely to be more vulnerable to COVID-19	**Yes**	**720 (79.2)**	0.76±0.43
	No	99 (10.9)	
	Not sure	90 (9.9)	
**Total knowledge level**		**86.08%**	

### Practice towards COVID-19

The mean practice score (±SD) of 13 questions was (21.56± 2.92). The correct percentage rate was 75.8%. We found that 92.1% of the participants started washing their hands frequently during COVID-19. Similarly, 92.1% indicated they had used sanitizer if the soap is not available, while usual handwashing with soap for 20 seconds was recorded by 77.2% of respondents. Almost 83.2% of participants wore a mask when they go outside the home. Three-quarters, 72.3% of respondents maintain space between themselves and others when going outside, and only 36.6% maintain the 2-meter distance between them and others to prevent transmission of the virus.

About 88% of the Iraqi respondents stopped going to crowded places recently. However, two-thirds of the respondents, 69.3% and 65.3%, reported that they stopped visiting and kissing their relatives or friends when meeting them, while 77.2% stopped the handshake during the greeting with others. Besides, 81.2% were aware of the essential of sanitizing their hands after using cash, and 75.2% of participants were aware of the importance of avoiding sharing their food with others. The participant’s practices toward COVID-19 prevention presented in Table 4.

**Table 4:** Practices of participants toward COVID-19in Mosul, Iraq (n = 909).

Practice questions		Frequency (%)	MEAN±SD
P1: Do you started to wash or sanitize your hands regularly?	Always	837 (92.1)	1.92±0.27
	Sometime	72 (7.9)	
	Never	0 (0)	
P2: Do you washing your hands for 20 Sec or more?	Always	702 (77.2)	1.54±0.84
	Sometime	0 (0.0)	
	Never	207 (22.8)	
P3: Do you use sanitizer if the soap is not available	Always	837 (92.1)	1.84±0.54
	Sometime	0 (0.0)	
	Never	72 (8)	
P4: Do you wear a mask when you go outside?	Always	756 (83.2)	1.81±0.43
	Sometime	135 (14.9)	
	Never	18 (2.0)	
P5: Do you keep distance between you and others when you go outside?	Always	657 (72.3)	1.72±.45
	Sometime	0 (0)	
	Never	252 (27.7)	
P6: Do you keep 2 meters distance between you and others?	Always	333 (36.6)	0.78±0.95
	Sometime	45 (5.0)	
	Never	531(58.4)	
P7: Did you stop going to crowded places recently?	Always	801 (88.1)	1.86±0.40
	Sometime	90 (9.9)	
	Never	18 (2.0)	
P8: Did you stop visiting your relatives and friends during the outbreak?	Always	630 (69.3)	1.59±0.66
	Sometime	189 (20.8)	
	Never	90 (9.9)	
P9: Did you stop kissing your relatives and friends when you meet them?	Always	594 (65.3)	1.58±0.62
	Sometime	252 (27.7)	
	Never	63 (6.9)	
P10: Did you stop handshaking with others?	Always	702 (77.2)	1.76±0.45
	Sometime	198 (21.8)	
	Never	9 (1.0)	
P11: Do you wash or sterilize your hands after dealing with cash?	Always	738 (81.2)	1.74±0.57
	Sometime	108 (11.9)	
	Never	63 (6.9)	
P12: Did you stop sharing your eating utensils and food with others?	Always	684 (75.2)	1.66±0.63
	Sometime	144 (15.8)	
	Never	81 (8.9)	
P13: Do you follow regular updates on COVID-19?	Always	693 (76.2)	1.73±0.51
	Sometime	189 (20.8)	
	Never	27 (3.0)	
**Total practices level**		**75.84%**	

### Sources of information on COVID-19

Participants indicated that the MOH in Iraq and social media such as Twitter, Facebook, YouTube, WhatsApp, Instagram, and Snapchat were the main sources of information about COVID-19pandemic with 60.3% and 57.5% respectively, followed by WHO press release 41.6%, while 28.7%, 15.8%, and 1.4% reported that they received their information from the news outlet, family and friends, and other sources, respectively as presented in Table 5.

**Table 5:** Sources of information related to COVID-19 reported by participants in Mosel, Iraq (n = 909).

Sources	Participant’s response (n)	Participant’s response (%)
Ministry of Health and Environment	549	60.30%
Social media	522	57.50%
WHO press release	378	41.60%
News outlet	261	28.70%
Family and friends	144	15.80%
Other sources	13	1.40%

### Level of knowledge (K) and practice (P) as per socio-demographic characteristics of participants

The knowledge level score (out of 15) showed a significant association across socio-demographic characteristics such as gender, age, education levels, marital status, and employment status (p <0.005). The practice level score (out of 26) also showed a significant association between gender and age-groups (p <0.005) while there was no significant association between marital status ((p = 0.061), educational (p = 0.385) and employment (p = 0.084) with the practice of the participants.

The results indicated that females had a higher mean score of knowledge (13.19±1.70) and practice (21.85±2.61) than males, aged group of participants above 50-years-old having the highest score of knowledge (14.11±0.87) and practice (22.50±2.32) compared with other age groups. Moreover, widows and divorced women’s knowledge (13.37±1.32) were higher than singles and married participants; however, there were no significant differences in practice. The mean score of knowledge (13.26±1.51) and practice (21.75 ±2.77) of participants with high education degrees were better than participants with lower educational degrees. Employed respondents showed a higher-level score of knowledge (13.12±1.61) than non-employed and students’ participants. While there were no significant differences in employment status in practice, neither education levels nor employment status had any significant differences in practice, as depicted in Table 6.

**Table 6:** Association between socio-demographic characteristics with knowledge (K) and practices (P) of participants.

**Knowledge of participants**					
**Variables**		**Frequency (%)**	**MEAN±SD**	**t/F**	**P-value**
Gender	Female	558 (61.4%)	13.19±1.70	6.57	0.000
	Male	351 (38.6%)	12.46±1.51		
Age	18–29	252 (27.7%)	12.35±1.63	64.54	0.000
	30–49	495 (54.5%)	12.80±1.70		
	≥ 50	162 (17.8%)	14.11±0.87		
Marital status	Single	288 (31.7%)	12.40±1.47	20.83	0.000
	Married	549 (60.4%)	13.11±1.74		
	Others	72 (7.9%)	13.37±1.32		
Education level	College degree	567 (62.4%)	12.84±1.62	9.28	0.000
	Postgraduate degree	234 (25.7%)	13.26±1.51		
	Others	108(11.9%)	12.50±2.07		
Employment status	Employed	585 (64.4%)	13.12±1.61	17.44	0.000
	Unemployed	198 (21.8%)	12.72±1.93		
	Student	126 (13.9%)	12.21±1.21		
Total knowledge score			12.91±1.67		
**Practices of participants**					
**Variables**		**Frequency (%)**	**MEAN±SD**	**t/F**	**P-value**
Gender	Female	558 (61.4%)	21.85±2.61	3.8	0.000
	Male	351 (38.6%)	21.1±3.31		
Age	18–29	252 (27.7%)	21.64 ±2.89	12.14	0.000
	30–49	495 (54.5%)	21.21±3.04		
	≥ 50	162 (17.8%)	22.50±2.32		
Marital status	Single	288 (31.7%)	21.87 ±2.87	2.80	0.061
	Married	549 (60.4%)	21.45 ±2.99		
	Others	72 (7.9%)	21.12 ±2.48		
Education level	College degree	567 (62.4%)	21.4 ± 3.07	0.955	0.385
	Postgraduate degree	234 (25.7%)	21.75 ±2.77		
	Others	108(11.9%)	21.73 ±2.36		
Employment status	Employed	585 (64.4%)	21.63±2.96	2.48	0.084
	Unemployed	198 (21.8%)	21.18±2.92		
	Student	126 (13.9%)	21.85±2.67		
Total practice score			21.56±2.92		

### Binary logistic regression analysis

Table 7 shows the binary logistic regression analysis on variables significantly correlated with (good and poor) knowledge and practice about COVID-19.

**Table 7:** Binary logistic regression analysis on factors significantly associated with mean knowledge and practices about COVID-19 of the participants, Mosul-Iraq.

**Knowledge (>12 and < 12)**			
**Variable**	**Β**	**OR (CI 95%)**	**P-value**
Age (> = 50)	RC	RC	
(18–29 vs. > = 50)	-20.859	1 (0.4–1.2)	0.995
(30–49 vs. > = 50)	-20.134	1(0.2–1.1)	0.995
Marital status (Others)	RC	RC	
(single vs. Others)	-0.924	0.397 (0.144–1.091)	.073
(married vs. Others)	-0.224	0.799 (0.334–1.910)	.614
Employment status	RC	RC	
(Unemployed vs. Employed)	-1.79	0.167(0.103–0.270)	0.000*
(Students vs. Employed)	0.397	1.487 (.826–2.677)	0.186
**Practice (>21 and < 21)**			
**Variable**	**β**	**OR (CI 95%)**	**P-value**
Gender (male vs. female)	-0.458	0.633 (0.464–0.863)	0.004*
Age (18–29 vs. > = 50)	-0.790	0.454 (0.261–0.788)	0.005*
Age (30-49vs. > = 50)	-0.890	0.411 (0.266–0.635)	0.000*
Education level (Others level vs. postgraduate	1.450	4.263 (2.206–8.236)	0.000*
Employment status (unemployed vs. employed)	-1.215	0.297 (0.158–0.558)	0.006*

* = p<0.05

RC = Reference category

The Odds ratios (ORs) and their 95% confidence intervals (CIs) in a bid to quantify the relationship between socio-demographic characteristics and the knowledge score (>12 and <12), and between socio-demographic characteristics and the practices score (>21 and < 21). Considering Odds ratios (ORs) equal to 1 shows no effect, an (ORs) greater than 1 shows variable increase the odds in good knowledge or practice, and odds less than 1 shows variables decrease the odds in good knowledge or practice [19]. Overall, the analysis presents a significant relationship between knowledge with age, marital status, and employment status.

The same table shows that the mean score of age ≥50 years reported better knowledge vs. other ages, no significant effect on the level of the knowledge has been observed within other ages. Similarly, Similarly, divorced & widows vs. married and single participants were significantly related with a higher mean score, with no significant influence on the knowledge level among married and single respondents. Employed people were significantly showed a better mean score of knowledge vs. unemployed and retired respondents, However, unemployed were less likely to present a good knowledge (β:-1.790, OR: 0.167, CI: 0.103-.270). Moreover, gender, age, education and employment status were significantly associated with good practices. Female gender reported a higher mean score of practices vs male, the regression analysis significantly showed poor practices in male gender than a female with odds ratio less than 1(β: -0.458 OR: 0.633, CI 0.464–0.863). Similarly, age group of 30–49 showed fewer commitments in the practices than other groups (β: -0.890 OR: 0.411, CI: 0.266–0.635). However, participants with a college-level degree and below could significantly increase the effect of good practices level comparing with the participants with a postgraduate degree (β 1.450 OR: 4.263, 95% CI: 2.206–8.236) while employment status of employed and students reported a better practice score than unemployed individuals, unemployed significantly showed poor practices than employed respondents (β: -1.215, OR: 0.297, CI: (0.158–0.558), as indicated in Table 7.

## Discussion

The public’s knowledge and practices play an important role in prevention by following the health guidelines to control the spread of COVID-19. The knowledge and practices of the general population about COVID-19have changed during the pandemic as a defense line against the disease.

The provision of baseline data regarding the level of individuals’ knowledge on clinical presentations, transmission, prevention, and risk factors of COVID-19 virus will help highlight malpractices related to preventive measures, making it critical for local authorities to plan suitable strategies to prepare and manage the spread of the virus.

The current study showed that the participants’ knowledge was high (86%) and had good practice measures toward COVID-19 (76%). Similar to our findings, several studies conducted in many countries have reported high levels of knowledge about COVID-19, among the general population in Malaysia 80.5%, Chinese residents 90%, Saudi Arabia population 81.5%, and among healthcare workers in Pakistan 93.2% [4, 14–16].

The high level of knowledge among the participants in this study may be because most participants have a college degree or higher, or due to the high level of media coverage, including all media outlets and the impact of the pandemic on social life mandating that people follow.

The current results showed that most of the participants depend more on the ministry of health and social media to get their information about the COVID-19, In contrast to other studies among Jordanian, Egyptian and Pakistani populations that were using mostly social media as the main source of information [20–22].

The study found that most respondents had a good level in prevention and control measures toward COVID-19 novel coronavirus, indicating that some respondents’ practices were very good toward COVID-19. That the results reported on practice toward COVID-19 among the respondents were similar to those reported in the Malaysian population [14] while being less than the Chinese residents’ level of practice [4].

This level of practice among the respondents attributed that the Iraqi government took drastic measures in reducing the spread of the disease and the low number of cases in Mosul at the beginning of the pandemic [23].

Our results using t-tests, ANOVA and logistic regression analysis showed that there were better knowledge and accurate practices associated significantly with the female gender, respondents above 50 years old, employed respondents, higher education, and married respondents.

Females and mothers show better knowledge and practices towards the COVID-19 precautions and preventions. Similar studies in Malaysian and Saudi Arabia indicated that females had more knowledge regarding COVID-19 than males [14, 15].

The high level of knowledge and practice among the participants above the age of 50 in our study is possibly due to understanding the higher risk of contraction and complications of the disease on the elderly and people with chronic diseases [24, 25]. In our study, respondents with a higher degree level had a greater knowledge level than the others. Similar findings were reported within Malaysian and Pakistani university populations [14, 22]. Majority of respondents confirmed that the COVID-19 disease is more dangerous in patients with chronic diseases and the elderly. This has been confirmed from many studies published regarding the COVID-19 in China [26, 27].

Our results reported that 17.5% of the respondents had chronic diseases such as diabetes, asthma, and severe obesity were the common diseases among our participants. A similar study among Iraqi adults indicated that the common non-communicable diseases were hypertension (13.3%) and overweight or obesity (54.6%). Another study showed that diabetes and hypertension were the most prevalent diseases among Iraqi people [27, 28].

Our results were compatible with many studies that showed similar significance in terms of better knowledge and practice of COVID-19 among the educated and employed people [4, 14, 15, 29, 30].

Finally, the results indicated that more intense health education efforts should be directed toward respondents with the following socio-demographic characteristics, male respondents, respondents with lower educational levels, younger respondents, and unmarried respondents.

### Limitations

Since the study is a cross-sectional study, it was conducted within a short time during the pandemic. Moreover, this study was an online survey that expected that the people with a higher level of education would respond to the survey; as such, it doesn’t give privilege to the uneducated population and those with limited access to the internet, and the results in this manuscript is not a representation of the total population of Mosul, Iraq. The study dataset was also self-reported, and this practice is subject to courtesy bias or social desirability bias.

## Conclusion

In general, the current study provided a comprehensive screening of the knowledge and practices of the among a sample of Mosul city population toward COVID-19. The participants had a high level of knowledge about the virus and good practice towards using protective measures, which is significant towards controlling the spread of the virus. The study recommends developing informative COVID-19 related campaigns targeted specifically towards those with the following socio-demographic characteristics; those below the age of 50, those single, married, younger males, and unemployed living in Mosul city.

## Supporting information

S1 Data (XLSX)

S1 Questionnaire (DOCX)
