# Supplementary material for: Socio-demographic correlate of knowledge and practice toward COVID-19 among people living in Mosul-Iraq: A cross-sectional study
Source: PLoS One. 2021 Mar 31;16(3):e0249310. doi: 10.1371/journal.pone.0249310 (PMC8011757; doi:10.1371/journal.pone.0249310)
Supplement: S1 Questionnaire — (DOCX) [file pone.0249310.s002.docx]

Study Questionnaire

**
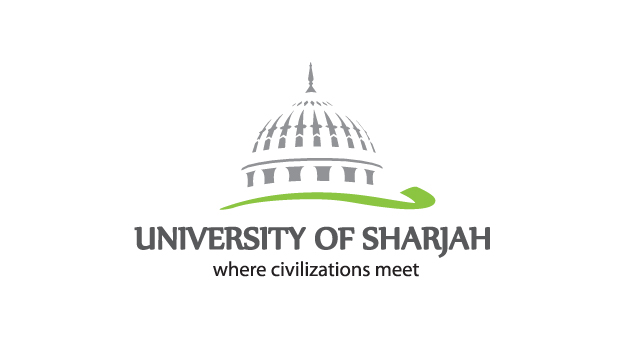
**

**Questionnaire on** **Socio-demographic correlate of knowledge and practice toward novel 2019 coronavirus among people living in Mosul-Iraq: A cross-sectional study**

**Demographic characteristics**

1. **What is your gender?**

- Male
- Female

1. **What is your age?**

- 18-29
- 30-39
- 40-49
- 50-59
- 60 and More

1. **What is your marital status?**

- Single
- Married
- Divorced
- Widower

1. **What Emirate do you live in?**

- Abu Dhabi
- Ajman
- Dubai
- Sharjah
- Fujairah
- Ras Al-Khaimah
- Umm Al Quwain

1. **What is your Education level?**

- Illiterate
- Primary
- High school/diploma
- College level
- Postgraduate (Masters/Doctorate)

1. **What is your employment status?**

- Full-time employment
- Part-time employment
- Unemployed
- Self-employed
- Retired
- Student

1. **Nationality**

- Emirati
- Non- Emirati

**knowledge about COVID-19**

1. **COVD-19 is caused by virus**

- Yes
- No
- Not sure

1. **Incubation period rang of COVID-19 is 2-14 day**

- Yes
- No
- Not sure

1. **The main clinical symptoms of COVID-19 are fever, dry cough, tiredness, and breathing difficulty**

- Yes
- No
- Not sure

### **Is there a vaccine for COVID-19?**

- Yes
- No
- Not sure

1. **Is there an active treatment for COVID-19?**

- Yes
- No
- Not sure

1. **COVID-19 is spreads via respiratory droplets (coughing, sneezing) of infected people.**

- Yes
- No
- Not sure

1. **COVID-19 is spreads via through touching contaminated surfaces**

- Yes
- No
- Not sure

1. **COVID-19 can be transmitted through eyes, in addition to nose and mouth**

- Yes
- No
- Not sure

1. **A person with COVID-19 having no fever cannot infect others.**

- Yes
- No
- Not sure

1. **Children and young adults don't need to take measures to prevent the infection by COVID-19.**

- Yes
- No
- Not sure

1. **We should stay at home and go out only when it is necessary**

- Yes
- No
- Not sure

1. **To prevent the spread of COVID-19, individuals should avoid going to crowded places if it's not necessary**

- Yes
- No
- Not sure

1. **COVID-19 may be more dangerous in patients with chronic diseases and elderly**

- Yes
- No
- Not sure

1. **People who have contact with someone infected with the COVID-19 virus should be immediately isolated in a proper place.**

- Yes
- No
- Not sure

1. **Smokers are likely to be more vulnerable to COVID-19**

- Yes
- No
- Not sure

**Practices during COVID-19 outbreak**

1. **Have you started to wash or sanitize your hands regularly?**

- Always
- Sometime
- Never

1. **Have you washing your hands for 20 Sec or more?**

- Always
- Sometime
- Never

1. **Do you use sanitizer if the soap is not available?**

- Always
- Sometime
- Never

1. **Do you wear a mask when you go outside?**

- Always
- Sometime
- Never

1. **Do you keep your distance between you and other when you go outside?**

- Always
- Sometime
- Never

1. **Do you keep 2 meters distance between you and others?**

- Always
- Sometime
- Never

1. **Did you stop going to crowded places recently**

- Always
- Sometime
- Never

1. **Did you stop visiting your relatives and friends regularly during the outbreak?**

- Always
- Sometime
- Never

1. **Did you stop kissing your relatives and friends when you meet them?**

- Always
- Sometime
- Never

1. **Did you stop handshaking with others?**

- Always
- Sometime
- Never

1. **Do you wash or sterilize your hands after dealing with cash?**

- Always
- Sometime
- Never

1. **Did you stop sharing your eating utensils and food with others?**

- Always
- Sometime
- Never

1. **Do you follow regular updates on COVID-19?**

- Always
- Sometime
- Never

1. **Where can you find more information about COVID 19? (You can choose more than answer)**

- Ministry of health and Environment
- WHO press release
- News outlet (Newspaper, Television, Radio)
- Social media (Face book, Instagram, Wats up)
- Family and friends
- Other sources………………….

5 (98.9)

3. I use alcoholic hand rub 555 (93.8)

4. I cough and sneeze in a ssue and throw it in waste bin 567 (95.8)

5. I wear a face mask 383 (64.7)

6. I drink ginger with honey 302 (51)

7. I eat garlic
